# Editorial: Biomechanics and mechanotransduction in cardiovascular calcification

**DOI:** 10.3389/fcvm.2026.1874143

**Published:** 2026-06-08

**Authors:** Joshua D. Hutcheson, Luis Cardoso

**Affiliations:** 1 Department of Biomedical Engineering, Florida International University, Miami, FL, United States; 2 Biomolecular Sciences Institute, Florida International University, Miami, FL, United States; 3 Center for Innovation in Cardiovascular Health, Florida International University, Miami, FL, United States; 4 Department of Biomedical Engineering, The City College of New York, New York, NY, United States

**Keywords:** biomechanics, cardiovascular risk, mechanobiology, modeling, vascular calcification

## Background to the research topic

Cardiovascular tissues operate in a uniquely demanding mechanical environment, continuously exposed to pulsatile pressure, flow-derived shear, cyclic stretch, and multiaxial deformation. These forces are not simply passive loads to be tolerated; they are biologically informative cues that shape cell phenotype, extracellular matrix (ECM) organization, tissue growth and remodeling, and ultimately disease progression (1). Across vascular beds and valves, mechanotransduction links hemodynamics and material behavior to inflammation, lipid handling, calcification, and structural failure (2, 3). Despite decades of progress, major translational gaps remain: (i) how to connect patient-specific mechanical environments to measurable biomarkers and imaging readouts; (ii) how to experimentally measure and connect multiscale structure–function relationships; and (iii) how to leverage mechanobiology to improve devices, replacement tissues, and durable therapies.

This Frontiers in Cardiovascular Medicine Research Topic, “*Biomechanics and Mechanotransduction in Cardiovascular Medicine*,” brings together five accepted manuscripts that collectively illustrate how cardiovascular biomechanics can be interrogated and operationalized—from imaging metrics that inform clinical decision-making, to engineered platforms that recreate disease-relevant mechanical cues, to direct measurements of how cyclic fatigue and calcification alter tissue integrity (Mendoza-Seale et al., Kostyunin et al., Jansen et al., Ferrières et al., Ran et al.). The studies span coronary artery disease, atherosclerotic plaque vulnerability, calcific aortic valve disease (CAVD), and bioprosthetic valve durability, highlighting both the breadth of mechanobiology and the recurring need to integrate mechanics with clinically actionable tools.

## Imaging and quantitative metrics that connect calcification to clinical interpretation

A persistent clinical challenge is that calcification—while mechanistically tied to disease progression—can confound diagnostic imaging and risk assessment. Ran et al. propose a practical approach to identify when coronary CT angiography (CCTA) can be trusted in the setting of calcified plaque. In a prospective cohort of 105 calcified stenoses with paired CCTA and invasive coronary angiography (ICA), they measured the Hounsfield unit (HU) of calcified plaque and adjacent blood and defined a ratio (RHu). RHu correlated with CCTA–ICA concordance (r = 0.509), with ROC analysis supporting a cutoff of 0.36: stenosis evaluation showed good concordance when RHu exceeded 0.36, whereas substantial bias emerged below that threshold. The authors further demonstrated feasibility *in vitro* and reported 74% prediction accuracy in a validation cohort. By connecting image interpretability to a simple, measurable attenuation relationship, this work underscores an important theme for the field: biomechanics-informed imaging metrics may help clinicians navigate artifacts (e.g., blooming/beam-hardening) that otherwise obscure the true lumen geometry in heavily calcified segments.

In a complementary direction, Ferrières et al. examine the inverse relationship between coronary calcification and vertebral volumetric bone mineral density (vBMD) measured from the same CT scan. In a retrospective cohort of 94 patients, those with a coronary artery calcium score of 0 had significantly higher vBMD (187.7 vs. 162.1 HU; *p* = 0.03), and this relationship persisted after adjustment for age, sex, BMI, and sedentary lifestyle. While motivated by the hypothesis that shared regulators might couple bone remodeling and vascular calcification, the authors did not find significant associations between calcification/vBMD and serum FGF23, sclerostin, or osteoprotegerin, concluding that these circulating factors in their dataset do not explain the observed relationship. Together with the Ran et al. study, this work reinforces the growing importance of quantitative CT-based phenotyping—not only for cardiovascular risk assessment, but also as a bridge to systemic disease relationships that likely intersect with mechanobiology and mineral handling.

## Microcalcifications, matrix organization, and plaque cap mechanics

One of the most compelling open problems in cardiovascular biomechanics is how microstructural heterogeneity translates into failure risk, particularly in atherosclerotic plaque caps, where microcalcifications may act as potent stress concentrators (4, 5). Jansen et al. address this challenge using a tissue-engineered (TE) atherosclerotic cap model incorporating hydroxyapatite (HA) particles to mimic microcalcifications across two size ranges (≤5 μm and ≤50 μm) and two volume fractions (1% and 5% v/v). They report that HA particles reduce mechanical properties under uniaxial tension, with notable decreases in stiffness and ultimate tensile stress (e.g., stiffness reductions on the order of ∼36%–43% across several conditions), supporting the notion that calcified inclusions can compromise cap integrity.

A particularly valuable aspect of this work is the explicit exploration of indirect, cell-mediated effects: the authors highlight that microcalcifications may not only alter local stress fields directly, but also influence tissue compaction and mechanobiological organization of the collagenous matrix, producing a reciprocal degradation in strength that could increase rupture susceptibility. This manuscript exemplifies an approach that the field increasingly needs—controllable experimental platforms that can be systematically varied to test hypotheses generated by computational models, while still preserving key biological features (cells, matrix remodeling, and time-dependent organization).

## Cyclic fatigue, hemodynamic environment, and structural deterioration of bioprosthetic valves

The long-term performance of implanted biomaterials and prostheses is also strongly shaped by their mechanical environment, including fatigue, wear, and host remodeling responses. Kostyunin et al. interrogate the drivers of structural valve deterioration (SVD) in bioprosthetic heart valves (BHVs), leveraging a unique paired-explant design: bioprosthetic mitral valves (BMVs) and tricuspid valves (BTVs) excised from the same four patients during reoperation for BMV failure.

Despite the prevailing view that chronic immune rejection is a major contributor to SVD, the authors observed that failing BMVs showed lipid deposition, hemorrhages, and tears consistent with mechanical incompetence, yet had *less* immune cell infiltration than their paired BTVs. Their findings support the interpretation that cyclic mechanical loading and fatigue are dominant drivers of failure in the higher-load mitral position. At the same time, immune infiltration—though present—may be secondary or context-dependent.

For the broader biomechanics community, this work emphasizes that “mechanical dose” (pressure, jet velocity, shear, deformation history) is not merely a background variable; it can reorganize the relative importance of biological processes (e.g., inflammation, cell adhesion, deposition) and thereby change how we should prioritize design constraints for next-generation BHVs.

## Mechanobiology-on-a-chip: controlled hemodynamics to probe early valve calcification

Finally, Mendoza-Seale et al. advance a microphysiological approach to mechanotransduction in CAVD using a CAVD-on-a-chip model of the valve fibrosa. They test the hypothesis that calcification-related readouts increase with endothelial cell presence, oxidized LDL (oxLDL) concentration, and physiologically relevant shear stress (20 dyne/cm^2^) over short-term (two-day) cultures. The authors report that endothelial cells and shear stress drive alkaline phosphatase activity, sulfated glycosaminoglycan production, and formation of multiple calcium phosphate species, including hydroxyapatite, with dynamic cultures revealing 3D cell–oxLDL interactions and associated ECM remodeling/endothelial dysfunction.

In addition to the biochemical and mineral outputs, they quantify endothelial membrane “pores” that increase with oxLDL and are exacerbated under shear, suggesting a mechanically amplified injury phenotype in this platform. As in the TE cap study, the key value here is controllability: devices like this allow the field to disentangle coupled variables (shear, cell–cell interactions, lipid oxidation) that are inseparable *in vivo*, while generating mechanistic hypotheses that can be tested in animal models and—ultimately—linked to patient phenotypes.

## Summary and future perspectives

Taken together, these five contributions reinforce a central message: cardiovascular disease is fundamentally a multiscale mechanics problem, and mechanotransduction provides the biological “wiring” that converts forces and material context into pathology. We anticipate that future progress will increasingly depend on linking patient imaging to validated, mechanism-driven models; integrating cell- and tissue-level biomechanics and mechanobiology with device and biomaterial design; and developing reproducible, quantitative endpoints that can move seamlessly from benchtop platforms to clinical cohorts. We hope this Research Topic serves as both a snapshot of current capability and a springboard toward mechanics-informed cardiovascular medicine.
